# Nanotechnologies in Obstetrics and Cancer during Pregnancy: A Narrative Review

**DOI:** 10.3390/jpm12081324

**Published:** 2022-08-17

**Authors:** Serena Bertozzi, Bruna Corradetti, Luca Seriau, José Andrés Diaz Ñañez, Carla Cedolini, Arrigo Fruscalzo, Daniela Cesselli, Angelo Cagnacci, Ambrogio P. Londero

**Affiliations:** 1 Breast Unit, Department of Surgery, DAME, University Hospital of “Santa Maria della Misericordia”, 33100 Udine, Italy; 2 Ennergi Research (Non-Profit Organisation), 33050 Lestizza, Italy; 3 Center for Precision Environmental Health, Baylor College of Medicine, Houston, TX 77030, USA; 4 Clinic of Obstetrics and Gynecology, University Hospital of Fribourg, 1752 Fribourg, Switzerland; 5 Institute of Pathology, DAME, University of Udine, University Hospital of Udine, 33100 Udine, Italy; 6 Academic Unit of Obstetrics and Gynaecology, Department of Neuroscience, Rehabilitation, Ophthalmology, Genetics, Maternal and Infant Health, University of Genoa, 16132 Genova, Italy; 7 Academic Unit of Obstetrics and Gynecology, IRCCS Ospedale Policlinico San Martino, 16132 Genova, Italy

**Keywords:** nanotechnology, nanoparticle, pregnancy, fetal therapy, preterm birth, preterm labor, preeclampsia, fetal growth restriction, fetal growth, diabetes, assisted reproduction technology

## Abstract

Nanotechnology, the art of engineering structures on a molecular level, offers the opportunity to implement new strategies for the diagnosis and management of pregnancy-related disorders. This review aims to summarize the current state of nanotechnology in obstetrics and cancer in pregnancy, focusing on existing and potential applications, and provides insights on safety and future directions. A systematic and comprehensive literature assessment was performed, querying the following databases: PubMed/Medline, Scopus, and Endbase. The databases were searched from their inception to 22 March 2022. Five independent reviewers screened the items and extracted those which were more pertinent within the scope of this review. Although nanotechnology has been on the bench for many years, most of the studies in obstetrics are preclinical. Ongoing research spans from the development of diagnostic tools, including optimized strategies to selectively confine contrast agents in the maternal bloodstream and approaches to improve diagnostics tests to be used in obstetrics, to the synthesis of innovative delivery nanosystems for therapeutic interventions. Using nanotechnology to achieve spatial and temporal control over the delivery of therapeutic agents (e.g., commonly used drugs, more recently defined formulations, or gene therapy-based approaches) offers significant advantages, including the possibility to target specific cells/tissues of interest (e.g., the maternal bloodstream, uterus wall, or fetal compartment). This characteristic of nanotechnology-driven therapy reduces side effects and the amount of therapeutic agent used. However, nanotoxicology appears to be a significant obstacle to adopting these technologies in clinical therapeutic praxis. Further research is needed in order to improve these techniques, as they have tremendous potential to improve the accuracy of the tests applied in clinical praxis. This review showed the increasing interest in nanotechnology applications in obstetrics disorders and pregnancy-related pathologies to improve the diagnostic algorithms, monitor pregnancy-related diseases, and implement new treatment strategies.

## 1. Introduction

Over the previous decades, significant advances in obstetrics have led to a reduction in mortality and morbidity associated with pregnancy complications [1,2]. Fundamental advances in neonatology have also ameliorated pregnancy outcomes and improved the survival rate of newborns delivered at earlier gestational ages, although not without an economic impact [1,3,4]. Fetal growth restriction, preterm birth, and cancer management during pregnancy still require research advancements to improve the quality of life of mothers and newborns, and possibly to reduce the costs of the postnatal management of newborns resulting from particularly problematic pregnancies.

For instance, the global average rate of preterm delivery was estimated in 2014 to be around 10.6% (9.0–12.0%) [5], representing a considerable concern in terms of morbidity (immediate and long-term sequels) and mortality [6,7]. Hypertensive disorders of pregnancy and preeclampsia are also potentially harmful pathologies [8,9], with a prevalence varying from 5% in low-risk to 20% in high-risk pregnancies [10]. Fetal growth restriction, which complicates approximately 10% of all pregnancies, alongside preeclampsia, is a contributing factor to preterm deliveries [11]. Gestational diabetes mellitus and pregestational diabetes mellitus significantly impact pregnancy, maternal, and offspring outcomes (e.g., favoring pregnancy complications, abnormal fetal growth trajectories, or long-term sequels) [12,13,14].

Furthermore, with the progressive aging of the female population and the childbearing delay [15], a significant increase in the incidence of pregnancy-associated breast (and other) cancers has been observed [16,17]. This increase boosts the demand for targeted and safe technologies that can also be used during pregnancy to improve the accuracy of diagnosis and the treatment of neoplastic pathologies.

The advancement of precision medicine strategies to manage these pathologies relies on a cornerstone that requires a more comprehensive understanding of the pathogeneses of these conditions, which is expected to lead to the implementation of current strategies and the development of new ones, with diagnostic and therapeutic applications [18,19]. As the science of engineering structures on a molecular level, nanotechnology offers a significant opportunity to achieve this goal [18,20].

Conceptually introduced in the 1950s, nanotechnology refers to the use of nanoscopic particles and devices with the potential to specifically integrate electronic, optical, fluid, and mechanical functions. In the biological field, these approaches are primarily applied in diagnostics and therapy [21,22]. The main purpose of nanotechnologies in the medical field is to improve the biodistribution, specificity, and targeting of bioactive molecules, in order to induce a desired therapeutic outcome while reducing potentially threatening side effects [22].

This review proposes a survey of the state of knowledge on the application of nanotechnology to obstetrics, focusing on its current and potential applications and safety. We will discuss the most recent discoveries in the early diagnosis, prevention, and treatment of maternal–fetal pathologies. Moreover, we will also highlight the issues associated with managing pregnancy-related malignancies, which represent a growing issue of our times, and provide insights on the role nanotechnology may play in developing intervention options.

## 2. Materials and Methods

A systematic and comprehensive literature assessment was performed, querying the following databases: PubMed/ Medline, Scopus, and Endbase. The databases were searched from their inception to 22 March 2022. Five authors independently screened the items and extracted those which were more pertinent within the scope of this review. The screened items which fall within the scope of this review should be peer-reviewed full-text articles discussing nanotechnology in the obstetric field. The following words were used in combination to identify the literature: pregnancy, pregnancies, gestation, pregnant, maternal–fetal, mother–fetus, mother fetus, obstetric*, tumor*, neoplasm*, neoplasia, neoplasias, cancer*, malignant, malignancy, malignancies, nanotechnology, nanomedicine, nanoparticle*, nanotherapeutic*, nanoformulation*, liposome*, micelle*, exosome*, nano theranostic*, nanofluidics, drug delivery, precision medicine, nano-obstetric*, and nanoobstetric*. Details about the queries are shown in Supplemental Table S1. In total, 11,982 items were found, with 7613 remaining upon the removal of duplicates. After the manual screening of the titles and abstracts, 674 items were considered relevant for this review, and were assessed for their full text. Selected articles included in this review are based on their relevance and scientific merit. The scientific merit assessment was based on full-text publication in peer-reviewed journals, excluding eventually retracted items. Meanwhile, relevance was based on the following principles: pragmatism to include the most valuable articles to give a comprehensive overview starting from literature reviews, pluralism to have as many perspectives as possible, and contestation to discuss conflicting data and debate arguments. A bibliometric investigation was also performed using data extracted from the Scopus database and saved in BibTeX format. The bibliometric analysis was carried out using the bibliometrix package (version 4.0.0) and R (version 4.2.1; R Core Team-2022. R: A language and environment for statistical computing. R Foundation for Statistical Computing, Vienna, Austria https://www.R-project.org/, accessed on 23 June 2022) [23,24]. The Scopus database was chosen because the references of each item were available, and it was possible to calculate a local citation ranking. We also assessed country collaborations, the keyword frequency over time, and word co-occurrences in titles and abstracts.

## 3. Nanotechnologies and Pregnancy

The use of drugs during pregnancy has increased significantly over time, probably due to the greater confidence that modern pharmacology has allowed us to acquire regarding the use of specific medication classes [25,26,27]. In spite of the greater awareness of medication use in pregnancy and lactation, information about indications and contraindications is not always available, especially in the case of newly introduced active principles or formulations. This paucity of information somewhat depends on the fact that trials seeking to ascertain drug effects on the fetus are performed solely in particular cases (e.g., treatments targeted explicitly for pregnancy or fetal in utero therapy), and pregnant women are willingly excluded in most studies due to ethical issues [28].

From this perspective, nanotechnology has only recently been applied to pregnancy and lactation, resulting in a new branch of medicine known as “nano-obstetrics”. Nanomedicine-based therapies are currently gaining great interest for what concerns the treatment of conditions affecting the mother, the placenta, and the fetus, and to improve the prognosis for both mothers and newborns [29]. A common fear of potentially damaging gametes, embryos, and fetuses, or even negatively impacting a woman’s reproductive potential, affects the development of innovative therapeutics in reproductive medicine and obstetrics. Modern delivery systems may provide alternative targeted intervention strategies by precisely targeting the source of the disease while minimizing short- and long-term consequences for the mother and the progeny [18,30,31].

Despite the evident success of nanotechnologies in the last few decades and the growing interest in the development of strategies which are able to temporally and spatially deliver drugs and retain them at the targeted site, pregnancy still represents a challenge for the application of these novel medications [31]. On the one hand, as introduced above, there is often a lack of data regarding the possible adverse effects on the mother and fetus, as for classical drugs [28,31]. Consequently, only phase IV observations allow us subsequently to draw definitive conclusions relating to any secondary effects in the female gender or specific conditions such as pregnancy. On the other hand, specific design and chemical features make nanoparticles capable of overcoming physiological barriers, such as the blood–placental barrier [32], and potentially to be active also on the fetus.

## 4. The Placental Barrier, Therapeutic Perspectives of Nanoparticles, and Nanotoxicology

During pregnancy, the blood–placental barrier regulates the transport of oxygen, nutrients, and residual products between the maternal and fetal bloodstreams. It also prevents fetal exposure to possibly harmful molecules from the maternal circulation. Therefore, the essential role that the blood–placental barrier plays in supporting the interaction between the mother and the fetus represents an interesting opportunity for the delivery of nanotherapeutics to treat various pathologies (e.g., targeting a drug specifically to the maternal compartment only, without entering the fetal compartment) [33].

During its intrauterine development, the fetus is vulnerable to chemicals and other substances that can impair its development [34]. A major limitation for the application of nanomedicine in the field of maternal and fetal health is the lack of established and reliable in vitro and ex vivo models which are available to adequately simulate the pregnant patient scenario [29]. Experimental models of the placenta have been developed to address the function of the placental barrier, which can be classified into in vivo, ex vivo, and in vitro models (Table 1) [35,36,37]. As expected, any of these models presents some strengths and some limitations. In vivo animal models can be costly and limited because of species differences [36,38]. Ex vivo explants and perfused placentae are optimal models of the placental interface but are unfortunately burdened with a short viability [36]. The static in vitro models based on cell cultures frequently use cancer cell lines with no variability, unlike in vivo models [35,36]. Recently, a better understanding has been promised by implementing new study models such as the placenta on-a-chip models, or organoids [35,36,37] (Table 1). The effect of nanoparticles on cell viability and biological barrier integrity has also been studied in many different epithelial barriers, such as the lungs, intestines, kidneys, and skin. All of this information can be helpful to better understand the interaction of nanoparticles with maternal and fetal tissues.

Nanoparticles can transit through the ordinary placental trans-cellular transport mechanisms such as pinocytosis, active transport, facilitated diffusion, and passive diffusion. The exact pathway is likely to be dependent on the particle size and the surface chemistry [36,39]. For example, gold nanoparticles could cross the placenta, arriving at the fetal circulation employing endocytosis, whether clathrin-mediated or caveolin-mediated [40]. Meanwhile, polystyrene nanoparticles were found to cross the placenta through passive diffusion [41]. Furthermore, polyethylene glycol-coated liposomes were shown to be mostly impermeable to the placental barrier [36,42]. Additionally, the CNKGLRNK peptide-coated liposomes specifically target the placental interface [43]. They do not pass into the fetal circulation, allowing therapies to be delivered right at the placental interface (i.e., the endothelium of the uterine spiral arteries and placental labyrinth) [43]. Various effects have been observed to depend on the chemical nature of the nanomaterial, nanoparticle size, surface coatings, and concentration, as well as on transepithelial electrical resistance and paracellular permeability [44]. Specific examples supporting the supposition that the modifications generated by specific nanomaterials on the placental barrier could lead to severe fetal growth impairment and development consequences are listed below.

High concentrations of polystyrene nanoparticles reduced in vitro the cell viability of choriocarcinoma cells (BeWo cell line) [45]. The authors attributed the effect to a previously known positive association between a high polystyrene dosage and a pro-inflammatory effect [45,46]. The in vivo administration of cobalt and chromium 80 nm nanoparticles resulted in neurodevelopmental abnormalities, and increased DNA damage in the fetal hippocampus [47]. Maternal–fetal oxygen transfer and the production of human chorionic gonadotropin were not modified by polyamidoamine dendrimer exposure in an ex vivo placenta perfusion experiment [48]. 

Another example is represented by silica nanoparticles, which reduced BeWo cell viability at high concentrations, possibly due to lipid peroxidation [44,49]. The application of transepithelial electrical resistance (TEER) could provide helpful information concerning the integrity of the epithelial cell layer, taking into account that its values before and after silica nanoparticle exposition did not change at low concentrations, suggesting that the barrier function remained undamaged [44,49]. Measuring TEER across a barrier is a non-destructive real-time method to assess barrier integrity [50]. In mice, an experiment showed that silica nanoparticles had significant adverse effects on the placental barrier, such as spiral artery impairment, blood flow reduction, and apoptotic cell death in spongiotrophoblasts [51]. 

Some authors excluded any short-term direct embryotoxic or teratogenic effects of quantum dots, whereas they observed that their long-term accumulation in the maternal organism might increase the risk of adverse effects on embryo development [52]. Furthermore, other authors excluded toxic silver accumulation in embryos/fetuses after the intravenous injection of silver nanoparticles in pregnant mice, despite a notable silver accumulation in the maternal liver, spleen, and visceral yolk sac [53]. This accumulation has been subsequently observed to affect embryonic growth and to delay physical and cognitive development in the offspring, likely through the induction of epigenetic changes in the embryo and the abnormal development of the placenta [54,55]. Consequently, perinatal exposure to silver nanoparticles should be limited or prevented in any case [56,57]. 

Nanoparticle permeability through the placental barrier may be affected by the disruption of tight junctions, which can compromise its physiological regulatory processes. Wang and coworkers demonstrated in an in vivo animal model that zirconium dioxide nanoparticles translocated through the placental barrier and accumulated in the fetal brain [58]. This process was mediated by receptor-based endocytosis and the tight-junction breakdown in the maternal and fetal blood placental barrier [58]. 

Different organic, inorganic, or hybrid nanoparticles have been previously tested (Table 2) [36,37,44,45,47,48,49,51,58]. Shojaei and coworkers reviewed the literature in detail on different placental models and the fetal risk assessment, considering various organic and inorganic nanoparticles [36]. Inorganic nanoparticles have been demonstrated to easily cross the blood–placental barrier and induce several toxicological effects [59]. In contrast, organic nanoparticles can be more selective in their target potential and show less toxicological effects [18,59]. However, additional studies are still required in order to broaden their application in the obstetric field [18,59].

Surface-functionalized nanoparticles can prevent transplacental passage and promote placental-specific drug delivery, thus enhancing medication safety and efficacy. Optimal results have been achieved, for instance, by combining nanoparticles with specific proteins which are exclusively expressed in the placenta, such as the placental chondroitin sulfate A-binding peptide or oxytocin receptor [60,61,62,63,64,65]. Among surface-functionalized nanoparticles, Zhang and coworkers proposed the use of placenta-specific exosomes as potential carriers for the placental targeted therapy of pregnancy complications [62]. The exosomes are extracellular vesicles with an endosomal origin physiologically produced by cells which are yet to be wholly characterized [66]. Their isolation, too, presents significant challenges, and the optimal methodology has yet to be developed [66]. Delorme-Axford and coworkers, in an ex-vivo study using human trophoblasts in primary cell cultures, found placental-derived trophoblastic exosomes to induce in non-placental cell defense against viral infections such as human cytomegalovirus by delivering exosomal miRNA (chromosome 19 miRNA cluster, C19MC) [67]. In two different time-series studies, the quantity and characteristics of exosomes through normal pregnancies were analyzed using maternal peripheral blood samples [68,69]. They found that placental-derived exosomes increase during the first trimester of pregnancy, and then gradually declined until delivery [68,69,70]. The same research group also found a differential release and function of exosomes in gestational diabetes mellitus [70,71]. Moreover, their therapeutic use is promising because they were found to be well tolerated after repeated treatments [66]. Furthermore, they are efficient at entering other cells with minimal immune clearance, and can be engineered to target specific cell types [66].

Other surface-functionalized nanoparticles can prevent transplacental passage and limit the exposure to the mother compartment. For example, doxorubicin is an anticancer agent that crosses the placenta and can harm the fetus. Soininen and coworkers demonstrated that polyethylene glycol-coated liposomes encapsulating doxorubicin exhibited, both in vitro (BeWo cells) and ex vivo (perfused a placental model), a lower placental permeability than the pH-sensitive liposomal formulation of doxorubicin and free doxorubicin [42].

Although the characteristics of nanoparticles could be predictive of their maternal, placental, or fetal uptake, the achievement of a comprehensive understanding of nanoparticle uptake, accumulation, and translocation, as well as of how their size, shape, surface chemistry, and charge affect biodistribution and therapeutic efficacy through predictive placental transfer models, is absolutely mandatory in order to determine how the timing and route of nanoparticle administration impact their distribution, effectiveness, and safety [72].

## 5. Point-of-Care Testing and Other Applications in Diagnostics

Along with therapeutic nanoparticles, novel nanotechnologies have been designed to improve diagnostic accuracy. Point-of-care testing is a medical concept to describe diagnostic testing at or near the point of care, which is the place and time of the patient care. This kind of advanced testing can be used in remote locations or outpatient facilities, reducing the costs of traditional medical laboratories, specialized operators, and complex equipment. One of the most prominent examples in obstetrics is the introduction of the home pregnancy test in the 1970s [73]. Through the years, the test has been modified, opening the way to the paper-based diagnostic technologies [74,75]. Generally, paper-based diagnostics, including lateral flow assays and microfluidic paper-based analytical devices, are affordable, user-friendly, rapid, robust, and scalable for manufacturing [74]. These diagnostics are optimal for the improvement of clinical pathways in remote settings and resource-limited areas. Nanotechnology was proposed to strengthen test performance in lateral flow assays or microfluidic paper-based analytical devices [74]. As previously mentioned, the introduction of a point-of-care test for pregnancy has modified the clinical management of early pregnancy, allowing the wide spread of the prompt detection of early pregnancy and the better planning of the following management. Moreover, using a β-HCG point-of-care test while assessing fertile women with low abdominal pain in outpatient facilities allows us, with low resources, to rapidly exclude from the possible causes of pain a life-threatening situation such as extra-uterine pregnancy. The widespread use of these types of test has changed clinical management, and has the potential to change clinical pathways further. Nanotechnology has recently been applied to human chorionic gonadotropin testing to improve detection sensitivity and widen the application of these tests in clinical management [76,77,78,79]. For example, Cai and coworkers assessed the possibility of using a nanotechnology-based test to detect β-hCG levels in peripheral blood and in uterine cervix secretions quantitatively [76]. In addition to the accuracy of the test, they proved that the cervical β-hCG/serum β-hCG ratio was predictive of spontaneous miscarriage and ectopic pregnancy diagnosis [76]. These advances can be essential in order to improve and simplify the diagnostic algorithms, which are standardized step-by-step procedures for reaching a diagnosis or management decision.

Nanotechnology-based approaches were also used to retrieve and isolate trophoblasts from the human cervix during pregnancy, allowing an early attempt at prenatal diagnosis starting from 5 weeks of gestation [80,81]. In particular, Bolnick and coworkers collected a cervical specimen with a brushing system for liquid-based cytology [80]. The cells were marked with an antibody specific for trophoblastic cells (anti-HLA-G) [80]. A secondary antibody conjugated with magnetic nanoparticles was used for the immunomagnetic isolation of the trophoblastic cells [80]. Other applications in the field of diagnostics include cell-free fetal DNA isolation for prenatal screenings [82,83,84,85].

Nanoparticles have been designed to improve imaging techniques which are traditionally considered off-limits during pregnancy. For example, the classical contrast medium of magnetic resonance imaging, gadolinium, is blamed for possible teratogenic and chromosomal damages. In order to overcome this issue, the use of superparamagnetic iron oxide nanoparticle ferumoxytol has been tested, with encouraging results and no impact at the maternal–fetal interface in pregnant rhesus macaques [86]. Moreover, liposomal gadolinium has also been proposed as a promising tool in the obstetrics field [87]. In particular, Shetty and coworkers found, in a mouse model, that liposomal gadolinium nanoparticles were confined to the maternal compartment without passing to the fetal compartment [88]. In addition, Badachhape and coworkers, in a mouse model, were able to visualize and study the retroplacental clear space through gestation using liposomal gadolinium nanoparticles [89]. Finally, microbubble contrast-enhanced ultrasound agents are also promising for the in vivo study of placental pathologies [90]. Even if microbubbles’ content and composition can vary, they are typically formed of an inert gas core stabilized by an outer shell assembled with lipids, carbohydrates, proteins, or polymers [90]. In particular, the advantage of microbubble contrast agents is their use in the study of the microcirculation (which is not accessible with ordinary Doppler-based studies) using an easy-to-use and cost-effective instrument such as the diagnostic ultrasound. Furthermore, it has no tissue toxicity, like gadolinium; meanwhile, it allows an adequate microcirculation study that can be useful in the imaging of the differential diagnosis of the malignant lesion without exposing the pregnant women to radiation or tissue toxicity.

## 6. Preterm Birth

The use of nanotechnology has been proposed for the treatment of preterm labor. This technology was suggested to improve the accuracy and cost-effectiveness of preterm delivery diagnosis by implementing a fibronectin test using magnetic nanoparticles coated with anti-fibronectin antibodies [91]. Furthermore, it was also applied to improve drug treatment. In the current practice, the use of drugs to reduce uterine contractions and prevent preterm birth is limited due to the systemic or fetal effects of currently available medications such as ritodrine or indomethacin [7,92]. Nanotechnology-based approaches have been developed to prevent the drug’s passage in the fetal bloodstream, and to target its localization at the uterine-wall level [60,61,62,63,64,65]. To this end, liposomes coated with antibodies or specific receptor antagonists were developed (e.g., placental chondroitin sulfate A-binding peptide, oxytocin receptor antagonist, or oxytocin receptor antibody) [60,61,62,63,64,65]. Moreover, nanotechnology was also used to improve the effectiveness of preterm-delivery-preventive drugs via the vaginal administration of progesterone, significantly reducing the prevalence of preterm birth in a mouse model and lengthening the time-to-delivery outcome by 39% [93]. In addition to the implementation of new devices for the administration of already known drugs, nanotechnology has also been exploited to optimize the delivery of innovative medicines, in order to counteract the mechanisms of inflammation known to be involved in preterm labor and offspring complications (e.g., improving the delivery of N-acetylcysteine) [94,95].

## 7. Preeclampsia and Fetal Growth Restriction

Both preeclampsia and fetal growth restriction are associated with aging and dysfunctional placentae [96]. In this field, nanotechnology was also applied with different intents: (1) to improve diagnostic management and (2) to implement treatment strategies. New nanotechnology-based point-of-care tests are being developed to improve the assessment of preeclampsia development risk [97,98,99]. For instance, recent advancements have been made in the development of electrochemical immunosensors for the early clinical diagnosis of preeclampsia within the 18–20th gestational weeks [100]. In particular, the aim would be to establish a high-specificity immuno-diagnostic platform which is able to detect and analyze multiple molecules simultaneously (at the picomolar resolution), which could be highly predictive for preeclampsia onset to anticipate and improve its further treatment. There is also a tremendous effort in the development of nanotechnology-based approaches to target the placental tissue in order to prevent or mild the consequences of preeclampsia and fetal growth restriction [101,102,103,104,105,106,107]. Another field of interest was the development of new disease-specific models [108]. In a recent article, Yu and coworkers precisely silenced a long non-coding RNA (lncRNA H19) in the placental tissue of a mouse model, obtaining in vivo the occurrence of pre-eclampsia-like symptoms [108]. This result is extremely important, and opens the path to future discoveries. First, the previous models of pre-eclampsia were mainly established by the systemic administration of drugs or surgery, hence inducing unwanted systemic toxicity and limiting the possible understanding of pre-eclampsia. Pre-eclampsia is supposed to be an obstetric pathology of placental origin [96]. Removing the placenta will bring back normal homeostasis in the mother, healing pre-eclampsia. This model lets us start pre-eclampsia from the placenta and not from a systemic interference, allowing us to understand the disease better. This result also means the possible development of new therapeutic strategies. In addition, this kind of model will allow the testing of possible etiopathogenic pathways in vivo, corroborating an eventual causative hypothesis.

## 8. Diabetes Mellitus

Nanotechnology has been proposed for the implementation of highly accurate tests for the diagnosis and monitoring of diabetes mellitus [109]. Recently, a nanotechnology-based point-of-care test was developed with the intent to monitor glycated albumin in gestational diabetic pregnancies [110]. In addition, different approaches (e.g., Cerium nanoparticles or zinc oxide resveratrol encapsulated in Chitosan) were also developed to treat gestational diabetes and its comorbidities [109,111,112,113]. For example, Du and coworkers found, in a streptozocin-induced diabetes rat model, that zinc oxide resveratrol encapsulated in Chitosan improves the diabetic biological signs, including a reduction in insulin resistance [112]. Moreover, recently in the same diabetes animal model, it was found that selenium nanoparticles could mitigate diabetic nephropathy during pregnancy [114].

## 9. In Utero Gene Therapy

In 2019, a viral-vector-based gene therapy for spinal muscular atrophy that delivers a functional copy of the survival of motor neuron gene cDNA was approved by the FDA. Since then, the interest in this field has grown [115], and nanoparticles have been exploited as carriers of nucleic acids in utero during embryonic or fetal life [116,117,118,119]. Although no human studies have been carried out until now for in utero gene therapy, many in vitro and in vivo studies have been performed [120]. In utero gene therapy is a potential game-changer for monogenic diseases because the treatment can prevent disease inception, avoiding early damage to the tissues. Another advantage is the prevention of immune system reactions to the gene therapy approach, limiting or overcoming its effectiveness, as it occurs in post-natal genetic therapy. Other potential benefits are the capacity to cross the blood–brain barrier or deliver the treatment with a high vector-to-target-cell ratio [120].

The broad container of gene therapy comprises endogenous gene editing, gene replacement or augmentation, and the use of antisense oligonucleotides to regulate protein expression [120]. However, these techniques bring some risks, such as insertional mutagenesis (observed in post-natal treatment) or the threat of germline transfer. In particular, insertional mutagenesis results from off-target gene insertion with the disruption of another biological pathway. An example of insertional mutagenesis was the development of T-cell leukemia several years after gene therapy for X-linked severe combined immunodeficiency in four patients [121]. In these four cases, the retroviral vector had inserted within the LMO-2 locus, which is known to be involved in T-cell leukemia development [120,121]. However, the potential is high, and preclinical studies focus on various pathologies such as spinal muscular atrophy, thalassemia, neuronopathic Gaucher disease, congenital retinal blindness, and cystic fibrosis [120].

## 10. Assisted Reproductive Technology

Another fascinating application field of nanomedicine related to pregnancy is assisted reproductive technology. With the progressive delay of first pregnancies due to contemporary social and working habits, modern society is now experiencing its highest infertility rate [15,122]. Novel techniques have been consequently developed to improve the conception rate in couples utilizing assisted reproductive technology. Exploring nanotechnology in non-human models is stimulating, as they make it possible to optimize newly developed protocols using nanomaterials against the impairments still faced by reproductive medicine [123,124]. For example, Abreu and coworkers used a non-invasive nanotechnology-based system to find the embryos with the highest chances of successful implantation among a pool of morphologically identical viable embryos [124]. In a preliminary study, they used an immunosensor to detect the embryo beta-HCG production, allowing improved embryo selection before implantation (the detection vs. no detection of beta-HCG was found to be a favorable prognostic factor for the establishment of a pregnancy) [124].

## 11. Cancer in Pregnancy

The increase of pregnancy-related cancer prevalence is a critical issue, partly due to the progressive delay of first pregnancies in high-income countries for work and social reasons [15]. Pregnant cancer patients represent a major concern in the modern maternal and fetal health field [125,126]. However, nowadays, many intervention options exist which can dramatically change patients’ prognosis, and consequently positively improve the therapeutic outcomes [19,127]. In most cases, patients require a personalized and integrated treatment which is necessarily drawn within a multidisciplinary setting in order to prevent the iatrogenic pregnancy complications which could be caused by cancer treatment, such as preterm delivery or impaired fetal growth [125].

The most frequent malignancy diagnosed during pregnancy is breast cancer [125,126]. Nanomedicine improvements can allow the safe administration of antiblastic agents during pregnancy, avoid improper therapeutic delays, and enable highly effective and safe treatment for the mother and the offspring [126]. An example is represented by the worldwide commercially available nanoformulations of paclitaxel, which are adopted against breast cancer due to their recognized favorable dosing regimens and low side effect profiles. However, transplacental transport and resultant fetal exposure to this nanoformulation remains an issue of debate, and ongoing studies are still drawing the future development of rational and safe treatment strategies for pregnancy-associated breast cancer and other perinatal diseases [128,129]. Additional formulations exist, such as polylactic glycolic-acid-encapsulated paclitaxel associated with Vitamin D3, liposomal doxorubicin, nanosomal docetaxel lipid suspension, or pegylated liposomal doxorubicin [42,126,130,131]. Recently, Ramaswamy and coworkers found that using, in a combination chemotherapy, the nanosomal docetaxel lipid suspension during pregnancy in a pregnant woman with fourth-stage breast cancer disease was safe and effective [126]. Liposome nanoparticles were also used to carry other drugs (e.g., NSAIDs or indomethacin) and limit their access to the fetal compartment [132]. Although rarer than breast cancer, other cancer types can also be diagnosed during pregnancy, such as hematological, skin, or ovarian cancer [125]. Recently, a review clearly outlined how nanotechnology has ushered in a new era of treatment in many different types of cancers [133]. Nanotechnology has allowed the improvement of pharmacokinetics, biocompatibility, and tumor targeting; the overcoming drug resistance; and the reduction of systemic toxicity [133].

In addition, several monoclonal antibodies have been developed that target the Her2 protein, expressed in several breast cancer patients, and with a notoriously poor prognosis [134]. The efficacy of these drugs in both the neoadjuvant and adjuvant setting has been one of the greatest successes of the last few decades against this frequent and life-threatening disease. Monoclonal antibodies represent a prime example of the most efficient modalities of targeted drug delivery we have today against malignancies, and probably represent the future of personalized medicine in the oncological field. Unfortunately, there is insufficient evidence to justify their use during pregnancy, which is why their administration is generally delayed in the puerperium. However, we can imagine that in the future, we will likely be able to offer monoclonal antibodies in formulations which are capable of sparing the fetus while battling the pregnant woman’s neoplastic disease. In fact, targeted liposomal carriers are an emerging field of research, and are capable of targeting a compartment or tissue specifically [63,64,65,132]. For example, Refuerzo and coworkers demonstrated in a mouse model that delivering indomethacin within multilamellar liposomes prevents the drug from passing the placental barrier, significantly reducing fetal exposure [132].

## 12. Bibliometric Analysis

**Figure 1:**
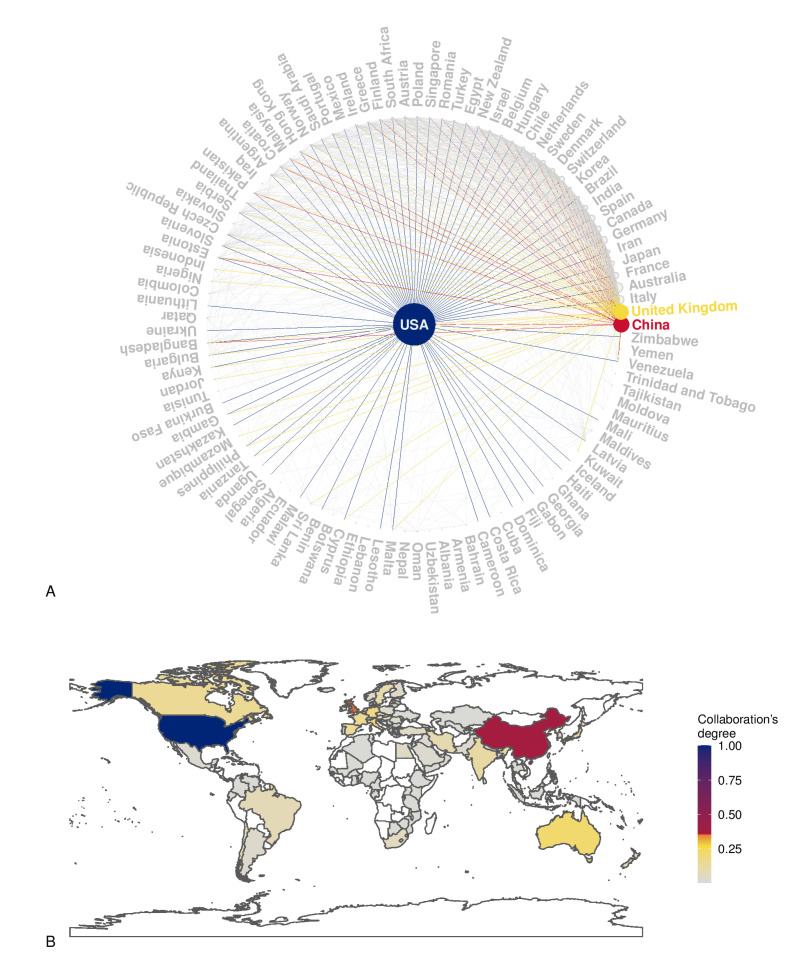
Panel (**A**): The plot shows the country collaboration network based on Scopus data. The vertex size is proportional to the number of collaborations, and the networks of the three most connected countries are highlighted. Panel (**B**): Spatial visualization of the degree of collaboration; blue and red colors correspond to the countries with more collaborations.

**Figure 2:**
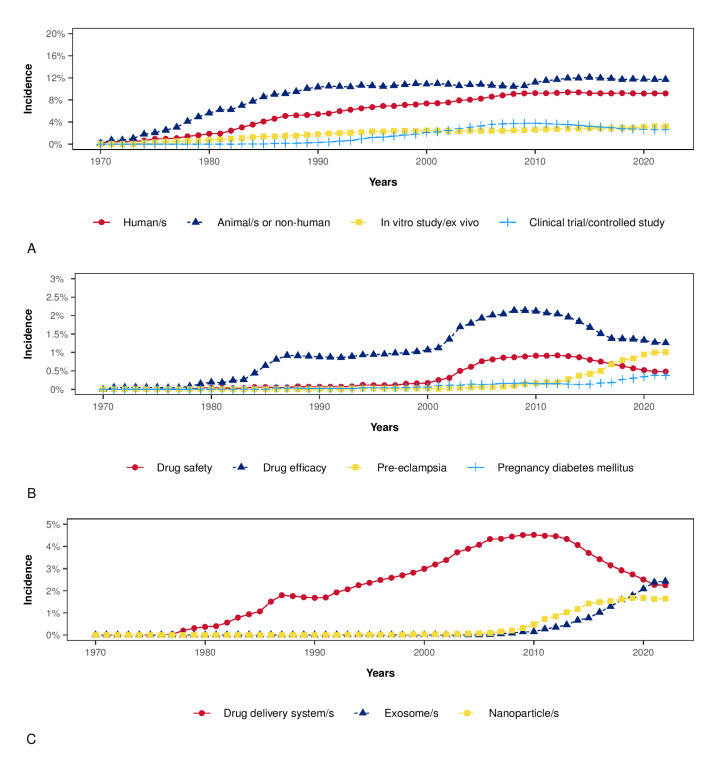
These plots show the annual incidence of a selected group of keywords among the top 100 (based on Scopus data). Panel (**A**): Keywords comprising the main type of studies. Panel (**B**): Keywords comprising the main topics in obstetrics. Panel (**C**): Trends in the annual incidence of the keyword “exosome” in comparison to “drug delivery system” and “nanoparticles”.

**Figure 3:**
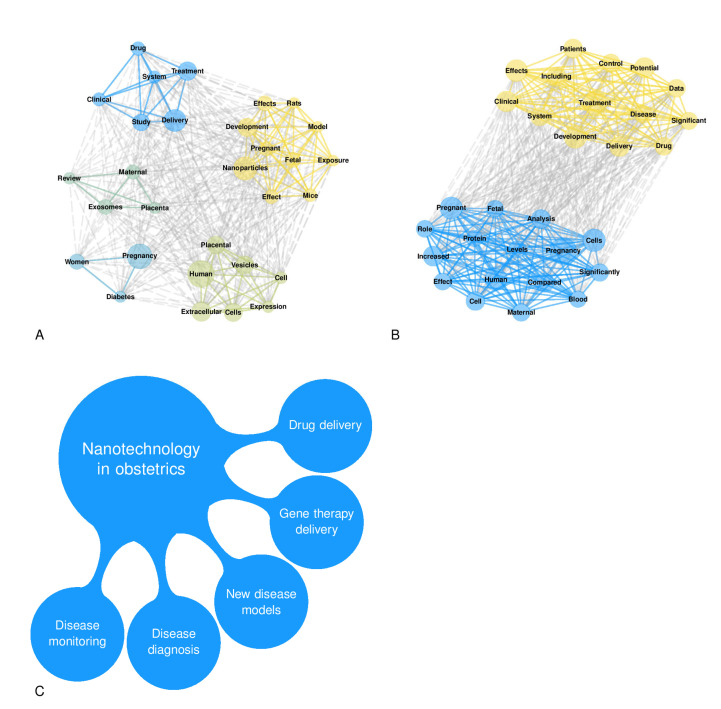
Panel (**A**): This network plot shows the title word co-occurrences from the Scopus database, considering the top 30 most frequent words. Panel (**B**): This network plot shows the abstract word co-occurrences from the Scopus database, considering the top 30 most frequent words. Panel (**C**): Main applications of nanotechnology in obstetrics.

A total of 4031 items were available in the Scopus database. The annual growth rate was 7.73%, the average number of citations per item was 26.55, the average number of citations per item per year was 3.30, and the total number of references cited by the 4031 included items was 230,112. Among the top five sources, two were obstetrics and gynecological journals (Placenta and American Journal of Obstetrics and Gynecology), and three were more generalist journals (International Journal of Molecular Sciences, Plos One, and Scientific Reports). Figure 1A,B illustrates the countries’ collaboration networks. The network shows active collaborations between high-income and low-income countries where some obstetrical pathologies’ prevalence is high, as are their detrimental consequences (e.g., Sub-Saharan Africa) (Figure 1B). Many efforts have been established in low-income countries to reduce maternal mortality and achieve the WHO Sustainable Development Goals [135]. Despite significant structural changes, still more work is needed in order to reduce maternal mortality due to pre-eclampsia and other obstetric complications [135]. The collaborations between high-income and low-income countries are essential in nanotechnology because of the possible advantages of the development of highly accurate and affordable point-of-care tests that can be cost-effectively implemented in a low-resource setting. Figure 2 shows the relative frequency of some keywords over the years. Figure 2A shows how the reference to humans has grown over time, as has the reference to controlled studies and clinical trials. However, the latter has slowed down over the last decade. At the same time, the reference to animal models is the most frequent, and has increased further in the last decade. Figure 2B shows the zenith of pharmacological safety and efficacy keyword frequency around 2010. Only since the end of the last decade has there emerged an increased reference to pre-eclampsia and diabetes in pregnancy (Figure 2B). Figure 2C shows the growing literature concerning drug delivery systems up to 2010; meanwhile, interest in exosomes has increased since then. Figure 3A shows the co-occurrence of words in the item titles showing five major clusters: one focused on drug delivery, one focused on fetal exposure, one focused on placental vesicles, one focused specifically on placental exosomes, and one focused on diabetes. Figure 3B shows the co-occurrence of words in the abstracts where two clusters were found, and one was focused on disease treatment. Among the top five most cited articles among the 4031 analyzed items in the Scopus database that were already discussed in this narrative review, three were about exosomes [68,69,70], one was about placental barrier capacity [32], and one was about metal nanoparticle toxicity [51].

## 13. Limitations

Despite this field’s tremendous interest and promises, nanotoxicity is the primary hindrance. Before using nanotechnology in the therapeutic management of pregnancy complications, more evidence should be collected in this field. However, it should be highlighted that this point is less limiting in diagnostic applications where there is no direct contact between the nanotechnology device and the pregnant patient, nor is there a risk of long-term adverse effects. A promising and emerging topic is the use of engineered exosomes as carriers. However, there are some limitations for the widespread adoption of exosomes. First, isolation technology should be improved. Second, their physiologic processes still need to be cleared entirely.

## 14. Implications for Clinicians and Policy-Makers/Healthcare Providers

The interest in nanotechnology is high because of the expected benefits to global society. The studies included in this review support these expectations, and we foresee growing support from the stakeholders for the expansion of research into the development of nanotechnology to improve healthcare in general and during pregnancy. Consequently, this increases exposure to nanomaterials, which also have potential toxic effects. For the foreseeable future, a universal set of guidelines for the nanotoxicity assessment of nanomaterials used in pregnancy by regulatory agencies will become mandatory in order to allow pregnant women to safely reap the benefits of nanotechnology-enabled products while assisting in the implementation of exposure controls to ensure maternal and fetal safety [136].

## 15. Unanswered Questions and Future Research

Nanotechnology in obstetrics is an evolving field, and the main suggested applications are shown in Figure 3C. In our opinion, the main impending questions to be answered in the future are presented in Table 3. In particular, we believe that the leading two open questions are the following: whether nanotechnology will allow for the early diagnosis (as soon as five weeks of gestation) of the major obstetrics pathologies and prenatal screening, and whether nanotechnology-based therapies will safely and specifically target the maternal compartment, the placenta, or the fetal compartment while minimizing therapy side effects.

## 16. Conclusions

In summary, this review showed the increasing interest in the application of nanotechnology-based strategies in obstetrics, such as improving the diagnostic algorithms, monitoring pregnancy-related diseases, and implementing new treatment approaches. However, their clinical application is yet to achieve maturity, despite the growing interest in the literature in the targeting of specific pregnancy pathologies in recent years. An emergent and promising approach is the use of exosomes to specifically target the therapeutic agents and avoid the toxicity of inorganic nanocarriers. Although the bibliometric analysis revealed a broad collaboration between high-income and low-income countries, in the future, the greater involvement of low-income countries would be desirable. Nanotechnology-based point-of-care tests have the potential to spread in low-resource settings, as beneficial instruments to improve the management of high-risk pregnancies and help to achieve the WHO Sustainable Development Goals Agenda.

## Figures and Tables

**Table 1:** Experimental models of the placenta.

Models	Advantages	Disadvantages
**In vivo**		
Mouse models	Pathological models Bio-distribution data Dynamic	Costly Species differences
Rat models		
**Ex vivo**		
Villous explants	Intervariability between samples Transplacental passage data Dynamic	Short viability
Perfused placenta		
**In vitro**		
Primary cell culture	Economical Primary throphoblastic culture with intervariability between samples	Only trophoblast cells Static model
Cell lines	Economical Well-known models	Only trophoblast cells No variability (tumoral immortalized cell lines) Static model
Placenta-on-a-chip model	Trophoblast and endothelial cells Economical Transplacental passage data Dynamic model	Low variability (tumoral immortalized cell lines to simulate maternal compartment)
Co-colture	Trophoblast and endothelial cells Economical Transplacental passage data	Low variability (tumoral immortalized cell lines to simulate maternal compartment) Static model
Organoids	Economical Transplacental passage data Dynamic model Symulate placenta development	Limited data available about placental barrier testing.

**Table 2:** Types of nanoparticles.

Types of Nanoparticles	Examples
Inorganic	Silver nanoparticles; gold nanoparticles; superparamagnetic iron oxide nanoparticles; cobalt and chromium nanoparticles; cadmium telluride nanoparticles; copper oxide nanoparticles; titanium dioxide nanoparticles; silicon dioxide; silica nanoparticles; zinc oxide nanoparticles; zirconium dioxide nanoparticles.
Organic	Dexamethasone-loaded polymeric nanoparticles; polyamidoamine dendrimers; polystyrene nanoparticles; carboxylate modified polystyrene nanoparticles; polyethylene glycol coated liposomes; polylactic-co-glycolic acid nanoparticles; fullerenes; liposomes nanoparticles; engineered exosomes.
Hybrid	Antibody conjugated with magnetic nanoparticles;liposomal gadolinium; superparamagnetic iron oxide nanoparticle; zinc oxide resveratrol encapsulated in Chitosan.

**Table 3:** Open questions.

Topic	Open Question
Diagnostic	Will nanotechnology permit the early diagnosis (as soon as five weeks gestation) of major obstetrics pathologies and prenatal screening? Can a cost-effective, highly accurate point-of-care test be implemented in clinical praxis in low-income and remote settings?
Placental models	Are the emerging placental models allowing a dynamic and accurate evaluation of the nanocarriers on the placental tissue, considering their distribution, accumulation, and toxicity?
Pathology models	Can nanotechnology-based animal models improve our knowledge about the pathophysiology of placenta-driven obstetric pathologies? Can emerging placental models improve our knowledge about nanotechnology’s effect on obstetrics pathologies driven by the placenta?
Treatment	Will nanotechnology-based therapies safely and specifically target the maternal compartment, the placenta, or the fetal compartment while minimizing therapy side effects? Will nanotechnology-based therapies improve the management of major obstetric pathologies such as pre-eclampsia, fetal growth restriction, or preterm delivery?

## Data Availability

All data were extracted from previously published studies, which are publicly available.

## Supplementary Materials

The following supporting information can be downloaded at: https://www.mdpi.com/article/10.3390/jpm12081324/s1. Table S1: Summary of database queries.
