# Supplementary material for: Nanotechnologies in Obstetrics and Cancer during Pregnancy: A Narrative Review
Source: J Pers Med. 2022 Aug 17;12(8):1324. doi: 10.3390/jpm12081324 (PMC9410527; doi:10.3390/jpm12081324)
Supplement: Supplementary file 1 [file jpm-12-01324-s001.zip › jpm-1782372-supplementary.pdf]

Journal of Personalized Medicine

MDPI

Supplementary Table S1. Summary of database queries.

Database	Query	Date	Number of Items
PubMed	(pregnancy OR pregnancies OR gestation OR pregnant OR maternal-fetal OR mother-fetus OR "mother fetus" OR obstetric*) AND (nanotechnology OR nanomedicine OR nanoparticle* OR nanotherapeutic* OR nanoformulation* OR liposome* OR micelle* OR exosome* OR Nanotheranostic* OR nanofluidics OR "drug delivery" OR "precision medicine" OR nano-obstetric* OR nanoobstetric*)	22.03.2022	6115
Scopus	TITLE-ABS-KEY(pregnancy OR pregnancies OR gestation OR pregnant OR maternal-fetal OR mother-fetus OR "mother fetus" OR obstetric*) AND TITLE-ABS-KEY(nanotechnology OR nanomedicine OR nanoparticle* OR nanotherapeutic* OR nanoformulation* OR liposome* OR micelle* OR exosome* OR Nanotheranostic* OR nanofluidics OR "drug delivery" OR "precision medicine" OR nano-obstetric* OR nanoobstetric*)	22.03.2022	4031
EMBASE	AB,TI(pregnancy OR pregnancies OR gestation OR pregnant OR maternal-fetal OR mother-fetus OR "mother fetus" OR obstetric*) AND AB,TI(nanotechnology OR nanomedicine OR nanoparticle* OR nanotherapeutic* OR nanoformulation* OR liposome* OR micelle* OR exosome* OR Nanotheranostic* OR nanofluidics OR "drug delivery" OR "precision medicine" OR nano-obstetric* OR nanoobstetric*)	22.03.2022	1836
